# Smaller Australian raptors have greater urban tolerance

**DOI:** 10.1038/s41598-023-38493-z

**Published:** 2023-07-18

**Authors:** Taylor Headland, Diane Colombelli-Négrel, Corey T. Callaghan, Shane C. Sumasgutner, Sonia Kleindorfer, Petra Sumasgutner

**Affiliations:** 1 College of Science and Engineering, Flinders University, Bedford Park, SA 5042 Australia; 2 Department of Wildlife Ecology and Conservation, Fort Lauderdale Research and Education Center, University of Florida, Davie, FL 33314-7719 USA; 3 Centre for Functional Biodiversity, School of Life Sciences, University of KwaZulu-Natal, P/Bag X01, Scottsville, 3209 South Africa; 4 Konrad Lorenz Research Center (KLF), Core Facility for Behavior and Cognition, Department of Behavioral and Cognitive Biology, University of Vienna, Fischerau 13, 4645 Grünau/Almtal, Austria

**Keywords:** Urban ecology, Behavioural ecology

## Abstract

Urbanisation is occurring around the world at a rapid rate and is generally associated with negative impacts on biodiversity at local, regional, and global scales. Examining the behavioural response profiles of wildlife to urbanisation helps differentiate between species that do or do not show adaptive responses to changing landscapes and hence are more or less likely to persist in such environments. Species-specific responses to urbanisation are poorly understood in the Southern Hemisphere compared to the Northern Hemisphere, where most of the published literature is focussed. This is also true for raptors, despite their high diversity and comparably high conservation concern in the Southern Hemisphere, and their critical role within ecosystems as bioindicators of environmental health. Here, we explore this knowledge gap using community science data sourced from eBird to investigate the urban tolerance of 24 Australian raptor species at a continental scale. We integrated eBird data with a global continuous measure of urbanisation, artificial light at night (ALAN), to derive an urban tolerance index, ranking species from positive to negative responses according to their tolerance of urban environments. We then gathered trait data from the published literature to assess whether certain traits (body mass, nest substrate, habitat type, feeding guild, and migratory status) were associated with urban tolerance. Body size was negatively associated with urban tolerance, as smaller raptors had greater urban tolerance than larger raptors. Out of the 24 species analysed, 13 species showed tolerance profiles for urban environments (positive response), and 11 species showed avoidance profiles for urban environments (negative response). The results of this study provide impetus to conserve native habitat and improve urban conditions for larger-bodied raptor species to conserve Australian raptor diversity in an increasingly urbanised world.

## Introduction

Urban landscapes act as a trait-based filter for wildlife, and responses to changes in environmental conditions may be influenced by species-specific phenotypic and behavioural traits^1–3^. Traits that generally promote positive responses to urbanisation include high fecundity, strong dispersal ability, behavioural flexibility, and increased tolerance and/or habituation to human presence^4–10^, but it is usually species dependent as to which traits are the most favourable^11,12^. Recently published literature shows that diet generalists tend to exhibit a positive response more often than diet specialist species in urban ecosystems^13,14^, as generalist species occupy broader niches that allow them to tolerate a wider array of landscapes^15–17^ and to explore a variety of different food resources^8,18^. As the world continues to urbanise^19,20^, understanding the species-specific traits that allow wildlife to survive within urban habitat is vital to maintain wildlife biodiversity.

Mechanistic responses, specifically of species in high trophic levels that fulfil a stabilising function in ecosystems such as raptors, have been largely overlooked. Raptors (species from the orders Accipitriformes, Cathartiformes, Strigiformes and Falconiformes) are apex predators that showcase archetypal examples of urban avoiders, adapters, and exploiters across the urban/rural gradient. Successful urban raptor species are traditionally bird specialist feeders^21–24^ due to the plentiful supply of food available for them in cities and towns, which allows them to be successful despite not fulfilling a generalist feeding niche that is usually associated with greater urban affinity^13^. Urban green spaces, such as parks, cemeteries and golf courses provide the habitat necessary for forest and woodland birds to forage, and raptors, such as the Peregrine Falcon (*Falco peregrinus,* pan-global), Cooper's Hawk (*Accipiter cooperii,* America), Black Sparrowhawk (*Accipiter melanoleucus,* Southern Africa) and Eurasian Sparrowhawk (*Accipiter nisus,* Europe), take advantage of these conditions^24–27^. Rodent specialist hunters and scavenging raptors are not uncommon within urban areas; however, their occurrence depends heavily upon prey availability^28–33^. As raptors are vital for ecosystem functioning through controlling prey populations and nutrient cycling^34^, prioritising feeding and breeding habitat for urban-tolerant raptor species is essential to enable biodiverse urban landscapes.

Urban raptors possess certain behavioural and phenotypic traits that enable successful breeding and foraging in urban ecosystems. Raptor home ranges encompass large areas, and urban centres may only be used to fulfil part of their ecological requirements (i.e., using urban areas for hunting, but more natural habitat for breeding or vice versa). Examples include Ospreys (*Pandion haliaetus*) that regularly use man-made structures (e.g. barges and platforms) for breeding, but feed almost exclusively on fish in neighbouring water bodies (e.g. rivers, estuaries, oceans, urban lakes and ponds)^35^, or Peregrine Falcons that breed on the cliffs of Table Mountain, South Africa, but use inner-city districts to prey on pigeons, doves and starlings^36^. The movement patterns of raptors are diverse, as some migrate thousands of kilometres to other continents^37,38^, while others are partially migratory^39,40^, or sedentary^41,42^. It is not clear how home range and movement patterns impact raptor urban tolerance, but sedentary birds show overall increased behavioural plasticity as opposed to migratory species^43^. Raptors that are capable of nesting on a variety of structures (e.g. trees and buildings) and raptors that exhibit flexible foraging techniques, such as perch hunting, pursuit and swoops^44–46^ or hunting under artificial light at night (ALAN)^47–49^, demonstrate adaptations that allow them to successfully survive in urban habitat by taking advantage of anthropogenic change^50^. Eurasian kestrels *(Falco tinnunculus)* in Slovakia have adopted a novel perch hunting technique that involves waiting above ventilation shafts to catch bats and common swifts (*Apus apus*)^51^, while Eleonora's falcons *(Falco eleonorae)* in Morocco are known to hunt migratory species disorientated by street lights at night^52^. The use of ALAN by raptor species in urban areas^48,53^ demonstrates that VIIRS night-time lights data are an appropriate proxy to study urbanisation patterns in bird species. Body size also plays a role in urban tolerance, as very small and very large raptors generally become extirpated from the urban environment^54,55^. This is likely due to a mixture of factors, namely tolerance to anthropogenic disturbance and suitability of the urban habitat for foraging and nesting^56^. However, there are exceptions to this, as very large scavengers (e.g. vultures) exist in urban areas where food availability is high and persecution is low due to socioeconomic, climatic, and biogeographic factors^32^.

Raptors continue to be understudied in urban areas, in part due to their sharp global decline^57,58^ and their general low population densities^34^, and thus high effort is required to conduct comprehensive studies. Community science is therefore an effective tool to assess raptor responses to urbanisation as it allows data collection over large spatial and temporal scales, utilising volunteers of differing skill levels to gather data across a variety of projects^59^. Projects such as eBird^60,61^ and iNaturalist^62^ amass millions of observations each year, and the data collected contributes to scientific publications or is used by various stakeholders, such as Government agencies and industry organisations^63,64^. Data from community science projects are invaluable in terms of time and effort, as these are generally the major limiting factors restricting researchers from collecting large amounts of data themselves^65,66^. Large datasets can also be challenging and time-consuming to analyse, often requiring copious amounts of data cleaning before analysis can commence^67^. Despite these limitations, data from community science projects continue to be a driving force behind scientific discovery, and growth in this sphere will exist as public awareness increases, programs expand, and technology advances^68–70^.

In this study, we used species occurrence data collected via eBird^60,61^, a global community science initiative documenting avian distributions worldwide, to assess the urban tolerance of 24 Australian raptor species and investigate whether specific phenotypic and behavioural traits, namely body mass, nest substrate breadth, habitat breadth, feeding guild, and migratory status, may explain species-specific responses to urbanisation. We predicted that species adapted to urbanisation (i.e. those with a positive urban tolerance index score) would be bird specialist or generalist feeders (e.g. feed on a variety of food types) rather than mammal specialist feeders, and nest on a variety of substrates allowing for more breeding opportunities^21^. We also predicted that urban adapters would be habitat-generalists and have a smaller to moderate body mass, as opposed to habitat specialists with a very large or very small body mass, as this pattern was previously found for raptors globally^54^. Our final prediction was that urban tolerant species would be sedentary species rather than migratory, as previous studies indicated that urban-adapted birds showed higher levels of sedentism, and some Australian species of raptors (e.g. Peregrine Falcon) are sedentary in Australia but migratory elsewhere^7,71–73^. As raptor research is largely biased towards a very small portion of the 557 raptor species, and the species with the highest number of publications (> 500) either have a pan-global distribution or are based in the Northern hemisphere, raising the profile of the conservation concern of Southern hemisphere raptors is a priority^58^. Based on the research and conservation prioritization index from^58^, Australia falls within the medium and high categories of the index within certain areas. Therefore, we tested these hypotheses in Australia, located in the Southern Hemisphere, to challenge current theories and assumptions that are largely based on raptor research conducted in the Northern Hemisphere^54,74^.

## Methods and materials

### Raptor observation data

We used observations of raptors across continental Australia from eBird^60,61^, a long-running community science project spanning the globe. Checklists of birds seen and heard are submitted by volunteer birdwatchers, along with user effort variables, such as survey duration, distance travelled, and spatiotemporal information, which are all recorded manually or by a phone application^75^. Since eBird began in 2002, users have submitted over 89 million checklists, amounting to over 1.2 billion observations of birds worldwide, making it one of the largest and most successful community science projects to date.

The eBird basic dataset for Australia (ver. ebd_rel_AU_Jun-2021; available at: https://ebird.org/data/download) was downloaded and all observations of raptors between 1 January 2010 and 30 June 2021 were used, as the vast majority of submitted checklists lie within this period (> 95%). As the aim of this study was to identify Australian raptor tolerance to urban environments at a broad temporal and spatial scale rather than examining changes between years, pooling the data over many years to include the largest amount possible was necessary to achieve this outcome. Checklists were filtered according to the eBird best practices guide recommendations^76^ to minimise the bias often present in community science datasets^77^. We filtered the data to include only ‘complete’ checklists—a case where the user had submitted a checklist of all the bird species they had seen/heard. Checklists that were ‘Stationary’ or ‘Travelling’ or followed Birdlife Australia survey protocols such as ‘Birdlife Australia 20 min-2 ha survey’, ‘Birdlife Australia 500 m radius search’ or ‘Birdlife Australia 5 km radius search’ were included, while checklists where the observer travelled for greater than 5 h or over 5 kms were removed to reduce observer variation effort^78^.

### Ecological traits

Ecological traits were selected from the existing literature that may influence avian tolerance to urban environments^54,56^. Data for body mass, nest substrate, habitat type, feeding guild, and migratory status were compiled from information found in the dataset ‘Biological, ecological, conservation and legal information for all species and subspecies of Australian bird’^79^, the books ‘*Birds of Prey of Australia: a field guide (3rd edition)*’^80^ and ‘Australasian Eagles and Eagle-like birds’^81^, and the online database ‘Birds of the World’ provided by the Cornell Lab of Ornithology^82^. Average body mass was used as a proxy for body size, and when possible, morphometric measures stemming directly from the Australian subspecies (e.g. Eastern Osprey) of a raptor were preferred from^79^ over other published material. Nesting substrate breadth categories were determined by searching the literature for all possible nesting structures that the birds may use and dividing them into six categories: building, other artificial structure (e.g. pole, barge, telecommunications tower), cliff, tree, water and ground. These values were then added to a total number of nesting substrate types recorded for each species. Habitat breadth values were calculated from^79^ by adding the total number of habitat types recorded for each species (Supplementary material A1). Feeding guilds were determined by examining the literature on species’ core diet and separating them based on four main categories: generalist (consumes a variety of food types), bird specialist, mammal specialist or fish specialist. Migratory status was classified as local dispersal or partially migrant, as there are no fully migratory raptor species in Australia^79,83^. We used the definition of local dispersal and partially migrant from^79^, and these definitions can be found in Table 1 in the ‘migratory status’ section.

**Table 1:** The traits used in the linear modelling analysis to investigate the association between traits and the urban tolerance index for each raptor species in Australia.

Trait	Description	Source
Body mass	The average body mass of the species. The value for the Australian subspecies was used where applicable	Garnett et al.^79^
Nest substrate breadth	Derived from 6 nesting substrates: building, other artificial structure (e.g. pole, telecommunications tower), cliff, tree, water, ground	Debus^80,81^, Billerman et al.^82^
Habitat breadth	Derived from 30 different habitat categories where species are known to feed; details provided in the supplementary material	Garnett et al.^79^
Feeding guild	Determined from primary food sources: generalist (consumes a variety of food types), bird specialist, mammal specialist or fish specialist	Debus^80,81^, Billerman et al.^82^
Migratory status	Local dispersal—taxa that are largely sedentary with dispersal by juveniles over small distances Partial migrant—taxa in which some individuals regularly move away from breeding areas after nesting but some remain behind all year	Garnett et al.^79^

### Measure of urbanisation

To quantify the relationship between species occurrence and the urban environment, we used VIIRS night-time lights^84^ data as a proxy for urban areas. It is a continuous measure readily available for download through Google Earth Engine^85^ that correlates positively with human population density^86,87^ and that is frequently used as a measure of urbanisation in ecological studies^13,88–90^. Whilst other measures of urbanisation exist^91,92^ (e.g. impervious surface cover, skyglow), we chose this method due to its ability to produce a continuous estimate that can individually rank species rather than placing species into arbitrary categories. Our choice was also driven by the fact that the available data existed mostly within the timeframe of this study at the appropriate spatial grain. The data product comes pre-filtered from sources of background noise such as degraded data, fires, and light source contamination for maximum precision. To obtain the median radiance value for each checklist, monthly rasters of the VIIRS night-time lights were combined from 1 January 2014 to 31 December 2020 to create a single raster in Google Earth Engine. This raster was imported into R^93^, where the median radiance was extracted within a 5-km buffer of each checklist. The ALAN median radiance values were condensed between 2014 and 2020 into a single value as exploratory analysis showed there were no large differences between years of a random sample of 1,000 distinct localities.

### Statistical analysis

Analyses were conducted using the statistical software R (v4.2) in the integrated RStudio environment^93^. The *tidyverse* workflow was used for data manipulation^94^, and the *ggplot2* package^95^ was used for figure plotting. To eliminate records where the birds were unlikely to occur and remove any unusual records, species checklists were cropped to the extent of their known ranges using shapefiles from the ‘Birds of the World’ dataset from Birdlife International^96^ using the *sf* package^97^, which is a common technique used within ecological studies^98,99^. Hexagonal grids of 5 km width were generated across mainland Australia using the *dggridR* package^100^ to facilitate spatiotemporal sub-sampling, a commonly used technique to remove potential spatial and temporal bias, as well as class imbalance (more non-detections than detections of focal species), within community science data^78,101^. Prior to modelling, one checklist was sampled from each grid cell from every week of the year across all available years (2010–2021) to remove any spatiotemporal bias, and detection and non-detection were sampled independently to deal with any class imbalance and ensure that not too many detections were lost. Exploratory modelling was then undertaken on all species; species under 1000 checklists with at least 1 observation produced large confidence intervals of their urban tolerance profile relative to the other species and were therefore excluded from the analysis. This reduced the initial set of 34 mainland Australian raptors to the final set of 24 candidate species for modelling (Supplementary material A2).

To examine urban tolerance in Australian raptors, generalised additive models (GAMs) were used with a negative binomial error structure to account for over-dispersion within the data. The eBird best practices guide^76^ was used as guidance for model preparation and fitting. The response variable for each model was the estimated abundance of each species within the checklist, while the predictor variable was the median VIIRS night-time lights value assigned to each checklist. Smoothing functions were applied to variables that were likely to influence the detection of a species on a checklist: number of observers, latitude and longitude, duration (min), day of year, effort distance (km) and ‘time observations started’. Thin plate regression splines were used for the variables: number of observers, latitude and longitude, duration (min), day of year, effort distance (km) with four degrees of freedom, and a cyclic cubic regression spline was used for ‘time observation started’ with 5 degrees of freedom. For each species’ model, the parameter estimate for night-time lights was obtained, indicating the relationship each species had with urbanisation (i.e. positive or negative) and the magnitude of that relationship. To reduce the uncertainty of the measure of urban tolerance due to the random sampling of eBird checklists within a grid cell, we ran our analysis 100 times for each species to obtain an average parameter estimate.

Multiple linear regression (i.e., all variables included in one model simultaneously) was used to investigate which ecological traits were associated with the species’ response to urbanisation, accounting for all other traits. The response variable was the species response to urbanisation (i.e. parameter estimate) extracted from the GAMs, while the predictor variable was the value of the five traits for each raptor (body mass, nest substrate breadth, habitat breadth, feeding guild, and migratory status) (Table 1). All quantitative predictor variables were scaled and centred prior to linear regression modelling, and visual inspection of residuals for model validation was undertaken.

## Results

**Figure 1:**
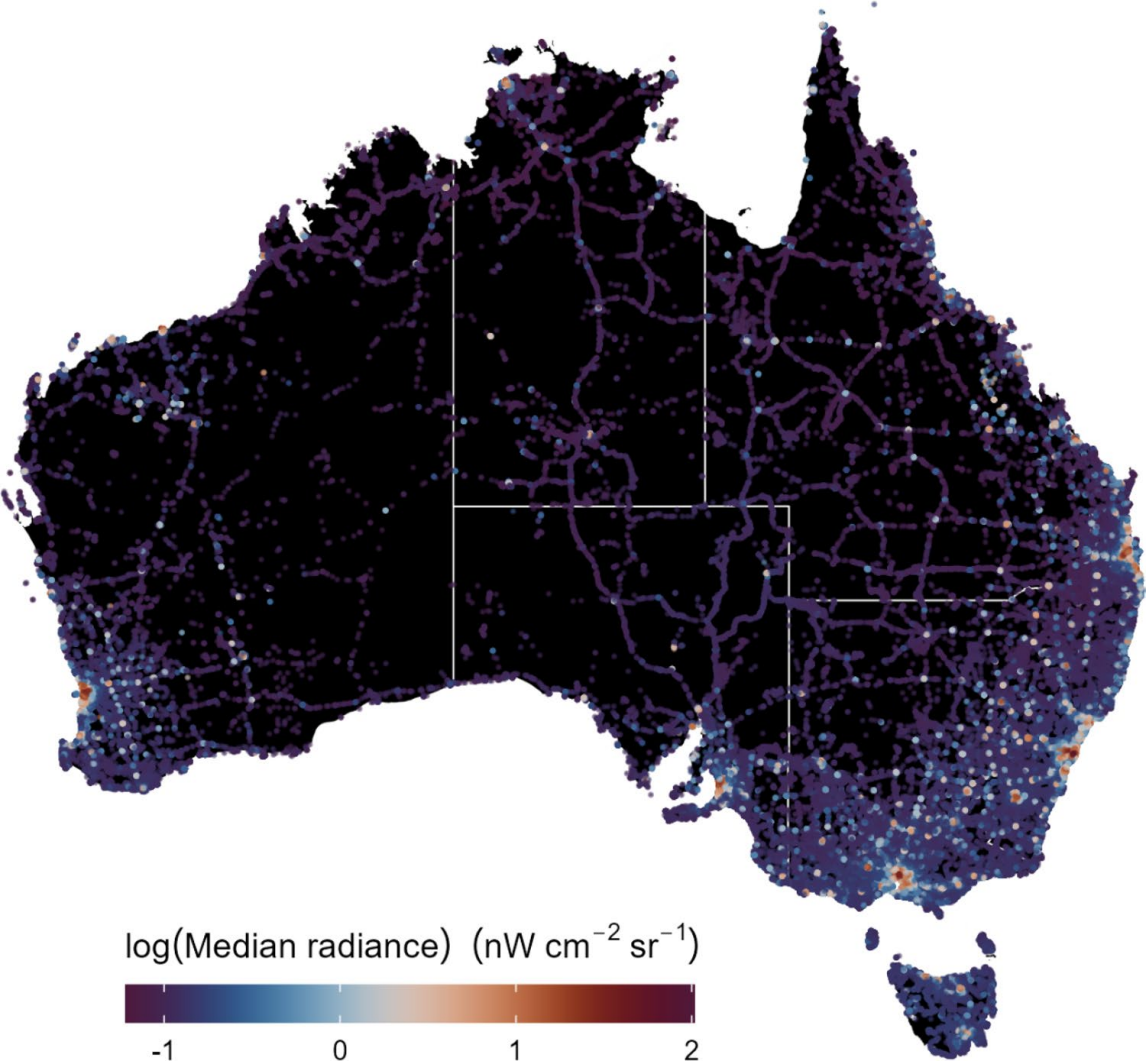
Map depicting the distribution of eBird checklists and their associated median VIIRS night-time lights value (log-transformed). Individual checklists are characterised by a coloured point, with purple and blue representing lower values and orange and red representing higher values.

A total of 840,918 eBird checklists were analysed, using 364,074 observations from 24 species prior to spatiotemporal subsampling, where one checklist was sampled across each 5 × 5 km grid from a species distribution range per week (Fig. 1). Spatio-temporal subsampling reduced the total number of species observations to 276,674. The Whistling Kite (*Haliastur sphenurus*) was detected the most of any raptor in the study, amassing 45,787 observations, while the Eastern Barn Owl (*Tyto alba*) was observed the fewest times, recorded on 1051 occasions across checklists (Supplementary material A2). Detection rates across sampled grids and the respective distributions of the study species can be found within the supplementary material (A3). The raptors observed in the area with the highest median radiance, or the brightest area across the study region, were the Brown Goshawk (*Accipiter fasciatus*) and Southern Boobook (*Ninox boobook*) (103.107 nW cm^−2^ sr^−1^) in Docklands Park, adjacent the Yarra River in central Melbourne, Victoria. A Whistling Kite was sighted in the area with the lowest median radiance (0.062 nW cm^−2^ sr^−1^), or the darkest area across the study region, which was at Lagoon Island, Lake Argyle, in north-eastern Western Australia.

**Figure 2:**
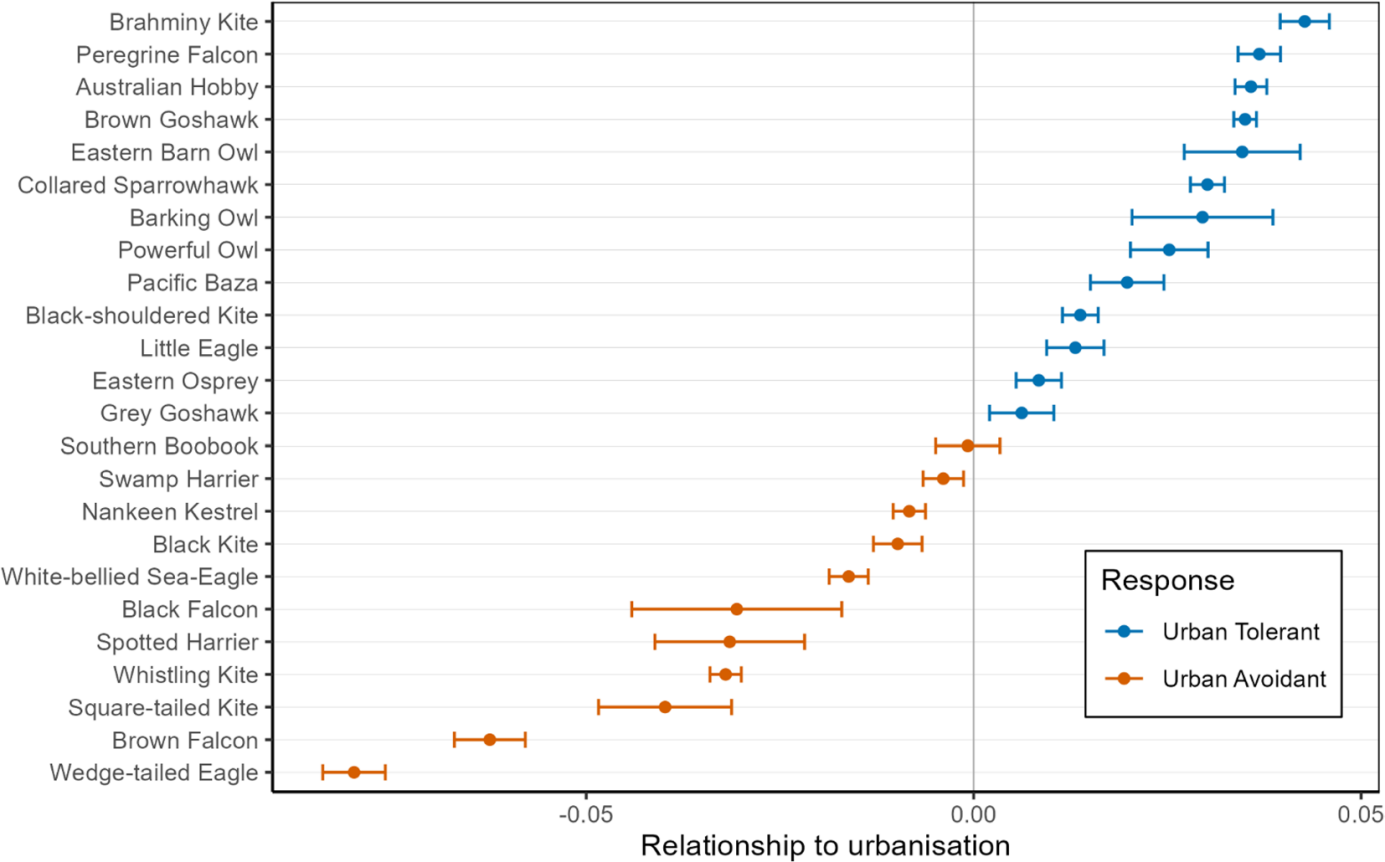
Urban tolerance index for the 24 Australian raptor species, ranked by the coefficient estimate from the generalised additive models. Larger positive values signify an increased effect of the predictor variable artificial light at night (ALAN), which indicates a positive response to urbanisation (‘Urban Tolerant’, in blue), while smaller negative values signify a decreased effect of ALAN as predictor variable, which indicates a negative response to urbanisation (‘Urban Avoidant’, in orange). The error bars represent the 95% confidence interval (of all 100 parameter estimates).

From the 24 raptor species included in the analysis, 13 species displayed a positive response and 11 species showed a negative response to urbanisation. The species with the highest tolerance to urbanisation were the Eastern Barn Owl and the Australian Hobby (*Falco longipennis*), while the Brown Falcon (*Falco berigora*) and the Wedge-tailed Eagle (*Aquila audax*) were the least tolerant raptor species to urban areas (Fig. 2).

**Figure 3:**
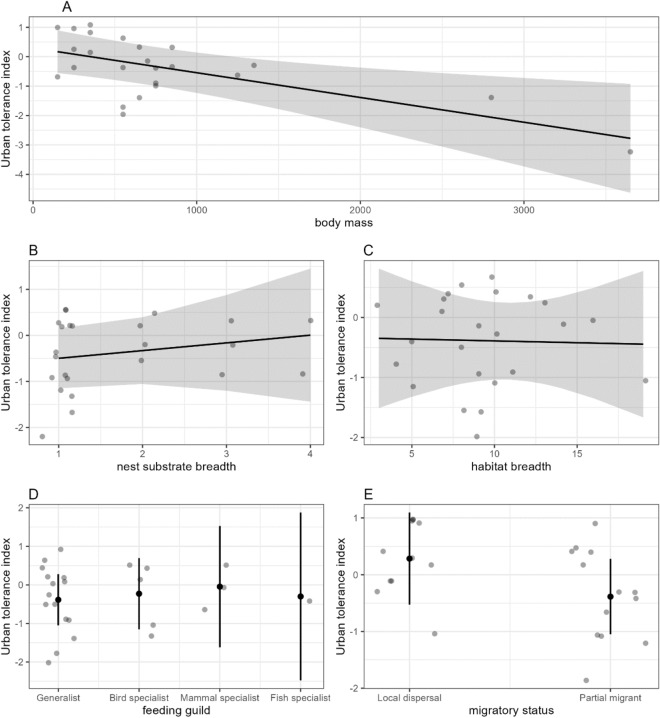
The Relationship between the urban tolerance index and ecological traits of 24 Australian raptor species. Marginal effects plots depict the relationship between urban tolerance and (**A**) body size, (**B**) nest substrate breadth, (**C**) habitat breadth, (**D**) feeding guild and (**E**) migratory status, accounting for all predictors. The grey points represent the partial residuals and the grey shaded area for (**A**), (**B**) and (**C**), and the black lines for (**D**) and (**E**) represent the 95% confidence interval.

A significantly negative relationship between raptor response to urbanisation and body mass was observed (Table 2), indicating that raptors with a smaller body mass (g) were more urban tolerant than larger-bodied raptors (F = 9.449, P = 0.007; Fig. 3A). No significant relationship was detected between the other variables and urban tolerance; nest substrate breadth (F = 0.559, P = 0.465; Fig. 3B), habitat breadth (F = 0.010, P = 0.920; Fig. 3C), feeding guild (F = 0.110, P = 0.953; Fig. 3D) and migratory status (F = 1.751, P = 0.204; Fig. 3E).

**Table 2:** Model summaries of the association between ecological traits of 24 Australian raptor species and their urban tolerance index for multiple regression linear modelling, including estimate, standard error (SE), t-value, lower and upper confidence limits.

Term	Estimate	SE	T-value	Lower confidence interval limit	Upper confidence interval limit
Intercept	0.284	0.383	0.742	− 0.528	1.096
Body mass	− 0.684	0.222	− 3.074	− 1.155	− 0.212
Nest substrate breadth	0.169	0.226	0.748	− 0.310	0.647
Habitat breadth	− 0.022	0.218	− 0.102	− 0.485	0.440
Feeding guild: generalist	–	–	–	–	–
Feeding guild: bird specialist	0.157	0.485	0.325	− 0.870	1.185
Feeding guild: mammal specialist	0.340	0.677	0.502	− 1.095	1.775
Feeding guild: fish specialist	0.085	1.025	0.083	− 2.088	2.258
Migratory status: local dispersal	–	–	–	–	–
Migratory status: partial migrant	− 0.670	0.506	− 1.323	− 1.743	0.403

The confidence interval is reported at the 95% level. The reference category for feeding guild was generalist, and the reference category for migratory status was Local dispersal.

Multiple r-squared—0.4413.

## Discussion

We assessed the urban tolerance of 24 Australian raptor species, whereby 13 showed a positive response to artificial light at night and 11 species showed a negative response. This finding highlights species-specific differences in urban tolerance across the Australian continent^13^, with some raptors showing tolerance response profiles in urban areas and others showing avoidance response profiles. Furthermore, body size was the main trait explaining the species-specific urban tolerance score, as smaller raptors were more likely to have greater urban tolerance index scores than larger raptors. Our results show the wide range in raptor tolerance response to urban environments, measured here using artificial light at night. Given that urban sprawl continues to develop across Australia, understanding the tolerance profiles of different raptor species to environmental change is vital information to inform conservation strategies for human-modified landscapes.

The Brahminy Kite (*Haliastur indus*) was found to be the most tolerant Australian raptor to urbanisation. Brahminy Kites are a coastal raptor, commonly seen soaring along the shoreline, as well as scavenging for food on beaches and jetties^80^. Records exist of Brahminy Kites breeding in urban areas, namely Darwin^102^, Northern Territory, and Port Macquarie^103^ and Port Stephens^104^, New South Wales, where there was varied breeding success across the study locations depending upon the level of human disturbance. A few factors may interplay to explain the tolerance Brahminy Kites to urbanisation, in particular its ability to breed on more than one substrate, its flexible diet and tolerance of human disturbance. Brahminy Kites are flexible in their breeding substrates, opting to use either large trees within mangroves or cities such as the African Mahogany (*Khaya senegalensis*) in Darwin^102^, or common artificial structures such as light towers^80^. Additionally, they also showcase a generalist diet which comprises of fish, birds, reptiles, crustaceans, amphibians, mammals, insects and offal^80,102^, which allows it to exist within a wide variety of different environmental conditions. The ability of Brahminy Kites to breed within urban areas highlights their capacity to tolerate human disturbance, but with increasing levels of urbanisation on the coast of Australia, there is an increased risk of poisoning from feral animal control and ingestion and entanglement from fishing equipment^103^. At the other end of the urban tolerance spectrum is the Wedge-tailed Eagle, the raptor with the lowest urban tolerance score. The species is known to be highly sensitive to human disturbance^105^ and to avoid urban landscapes. For example, human activity from mountain bikers, off-road vehicles and bushwalkers has the potential to impact breeding success in Wedge-tailed Eagles that are located close to urban areas in Perth, Western Australia^106^. Wedge-tailed Eagles will retreat from urban expansion^107^, however, some individual pairs show a higher disturbance tolerance to human activity when breeding inside protected reserves^108^.

The finding that larger raptors have lower urban tolerance than smaller species is consistent with findings from other studies investigating urban raptor occurrence^54,55^. One particular study undertaken in Reno-sparks, Nevada, USA, showed that Golden Eagles (*Aquila chrysaetos*) breed the furthest away from urban development when compared to other smaller species, and the authors concluded that habitat requirements (e.g. large, open terrain) and life history traits (e.g. small clutch sizes, long-post-fledging dependency) likely explained this result^109^. In our study, Australia’s largest birds of prey, the Wedge-tailed Eagle, and White-bellied Sea-Eagle (*Haliaeetus leucogaster*), were both found to avoid urban areas. Given that body size usually correlates with life history ‘speed’^110^, this negative correlation between urbanisation and eagle occurrence might have a similar explanation to the one reported for Golden Eagles^108,111^. Wedge-tailed Eagles usually nest several kilometres away from human developments^105,108,112^, while White-bellied Sea-Eagles can occasionally nest within urban green space^113^ using forested zones scattered throughout the metropolitan area^114^. However, from a global perspective, larger raptors are not always urban avoiders: in South Africa, for example, Crowned Eagles (*Stephanoaetus coronatus*) feed on urban exploiters such as the Rock Hyrax (*Procavia capensis*), Hadeda Ibis (*Bostricia hagedash*) nestlings, and Vervet Monkeys (*Chlorocebus pygerythrus*) which support a large urban breeding population of Crowned Eagles in Durban and Pietermaritzberg^115^. In Vancouver, Canada, Bald Eagles (*Haliaeetus leucocephalus*) feed on a variety of birds and fish, and commonly nest in tall Black Cottonwood (*Populus trichocarpa*) and Douglas Fir (*Pseudotsuga menziesii*) trees, occasionally choosing to nest on transmission towers^116^. A metanalysis of 172 threatened and near threatened raptors around the world identified body size as the strongest predictor for their conservation status^117^, whereby the larger the species, the higher the potential for exposure to anthropogenic threats and conservation concern. This association between body size and conservation status highlights the need to safeguard suitable habitat outside of cities to meet the requirements for large raptor species in the future.

In Australia, raptors with smaller body mass (172 g to 370 g) were generally tolerant of urbanisation, while medium-sized raptors (548 g to 847 g) displayed a variable response (e.g. tolerant or avoidant) to urbanisation. A potential driver of this trend may be the distribution of suitable prey residing within and outside urban areas, which can be linked to body size. Avian specialists are known to thrive in urban areas^21^, as they profit from an increased density of avian prey attracted to supplementary food sources such as bird feeders^118,119^, which are a common feature amongst Australian Gardens^120,121^, and large numbers all-year-round of starlings, doves and pigeons^35^. Many of Australia’s bird specialist feeders have a smaller body mass [e.g. Australian Hobby, Peregrine Falcon and Collared Sparrowhawk (*Accipiter cirrocephalus*)], enabling for swift pursuits of their avian prey. Australian cities include a mosaic of vegetation that is likely to attract birds^122,123^. This includes *Eucalyptus* spp. that are suitable nesting trees for both large and small raptors in Australia^81,124^, and the urban remnant bushland^125,126^, as well as exotic shrubs and flowers planted in gardens^127^, that can provide nectar all year round^128^ for species such as honeyeaters and parrots^129^ upon which raptors can feed on. Many of the raptors with a moderate body mass are diet generalists, such as the Brahminy Kite and Spotted Harrier (*Circus assimilis*). These species displayed markedly different urban tolerance profiles, which could be a function of the distribution of their prey existing either inside or outside of urban habitat. However, habitat preferences may also play a role in this phenomenon, and therefore further research is needed to clarify the link between Australian raptors of medium body size and urban tolerance and the underlying mechanisms driving the pattern.

Partially migrant and sedentary species had similar urban tolerance profiles, which is consistent with the findings from recent studies focussing on raptors across the globe^54^ and in Argentina^130^. Little Eagles (*Hieraaetus morphnoides*) are partially migratory, usually migrating from Southern Australia to Northern Australia during the winter months^131^. Ongoing GPS tracking studies have confirmed that the habitats used by breeding Little Eagles in Canberra were similar to those used during migration (woodland, grassland, forested areas, open urban land), and they appear to be tolerant of human activity and urban landscapes regardless of their breeding or migration state^132^. Booted Eagles (*H. pennatus*), a close relative of the Little Eagle, also showed positive responses to urban landscapes, as a population increase in western Europe was observed due to an increase in suitable prey^133^. Ongoing monitoring of raptor migration will be important to locate key areas used by urban-adapted species, potentially also as suitable stop-over spots during migration, to ensure their conservation.

### Study limitations

While large-scale data collection by community scientists can facilitate continental-wide data, we acknowledge that such data face several limitations. For example, owls are nocturnal hunters, well camouflaged and cryptic in nature, which results in a lower detectability that often relies on identification by call rather than a visual confirmation. Sightings of owls may be more biased towards brighter urban areas, as artificial light sources such as streetlights and industrial lighting could enable easier observation. A clustered detectability may be apparent because of known roost sites, and in combination with some observers (i.e. birders) keen to take advantage of ticking off a target species, can lead to an over-representation of one single individual in an area^134^. We also recognize that most of the Australian population lives coastally, and therefore checklists are heavily biased towards these areas and along main highways connecting inhabited regions. Even though spatiotemporal sub-sampling was used to mitigate such biases, such clustering of observations still occurred, especially in data rich areas. But, as raptors were the only taxa investigated in this study, which are usually detected using the same methods and the observations are subject to the same biases, it is probable that the systematic sampling bias is analogous for all species observed in this study^13,135^.

ALAN was used as a continuous metric of urbanisation within this study, and whilst this measure of urbanisation correlates positively with human population density and impervious surface cover^136,137^, urbanisation occurs across large spatial scales, from the landscape to the local level^138^. Therefore, it is likely that across these scales species responses to urbanisation may differ^139^, and the results from this study reflect Australian raptor responses to urbanisation at a broad scale rather than a fine scale, with the limitation that ALAN was used as a proxy for urbanisation. However, while ALAN is a proxy for urbanisation, it could also serve as a sensory pollutant for raptors, impacting the biological clocks of raptors and their prey. For example, owl species in this study could use night-time lighting as artificial hunting hot spots where prey may congregate to the lights, whereas larger species such as eagles may avoid well-lit areas due to their sensitivity to anthropogenic disturbance. To assess urban tolerance more accurately at finer scales, rather than the broad-scale approach like we have used here, data from GPS-tracked birds or survey data assessing the occupancy of birds in urban areas in conjunction with high-resolution landcover data would be a more suitable approach. Further, the results showed that body mass was the only trait that significantly influenced urban tolerance in Australian raptors, and no other traits influenced urban tolerance. The non-significance of the other traits may have been because of the coarse resolution that the traits were selected at (e.g. continental Australia). To be reliable, generally functional traits need to be location and individual specific^140^, however when working at the macroecological scale and assessing interspecific differences, coarser trait resolution is suitable^141^. As we were assessing tolerance at the landscape level, we chose to select traits at a coarse scale as it was the most useful resolution for this study, but we acknowledge that the reliability of these traits across time and space for some species may be significantly decreased.

### Future areas of study

The eBird checklist numbers in Australia are growing more numerous each year, and therefore investigations into the urban tolerance of raptor species that occur at lower densities (e.g. Red Goshawk) may become feasible in the future, most likely in conjunction with targeted surveys from conservation related organisations. Also, a more granular examination of habitat use within urban areas of urban tolerant raptors will be an important area of future research to conserve important foraging and breeding areas. Such approaches will help identify which raptor species are occupying urban areas during the breeding season, and those that only visit to forage or roost.

## Conclusion

In summary, this research used a large continent-wide raptor data set collected by community scientists and professional birders across Australia to generate valuable insights into the urban tolerance of 24 Australian raptor species. The finding that the 13 species with greater urban tolerance also had, on average, smaller body size, sheds light on mechanistic pathways that may be driving urban tolerance response profiles. Smaller-bodied species tend to have faster life histories and higher metabolic rates, producing larger clutches earlier in life that are frequently provisioned with relatively small prey. The abundance and commonality of nocturnal and diurnal prey including small mammals, rodents, pigeons, doves, and passerines, in conjunction with the diet speciality of many small Australian raptors, may favour the persistence and survival of smaller-bodied raptors in urban environments. Conservation management initiatives, particularly those that focus on habitat preservation and restoration (e.g. wilderness area protection), are needed with a special focus on protecting larger-bodied raptor species given urban expansion and an avoidance response of larger raptor species to urban areas.

## Supplementary Information

Supplementary Information.

## Data Availability

The data and code to reproduce these analyses are available here: 10.5281/zenodo.8093060.
