# Supplementary material for: Smaller Australian raptors have greater urban tolerance
Source: Sci Rep. 2023 Jul 18;13:11559. doi: 10.1038/s41598-023-38493-z (PMC10354083; doi:10.1038/s41598-023-38493-z)

Supplementary material

Smaller Australian raptors have greater urban tolerance

Taylor Headland, Diane Colombelli-Négrel, Corey T. Callaghan, Shane C. Sumasgutner, Sonia Kleindorfer, *Petra Sumasgutner

A1. Derivation of traits from Garnett et al. 2015

The dataset from Garnett et al. 2015 provides morphological, ecological and general biological information for all Australian species and subspecies of birds. Habitat breadth, migratory status and body mass were calculated from this dataset. Habitat breadth and migratory status values were used at the species level, but if body mass was available for the Australian subspecies, that was used in its place.

Below is a description on the value and the column(s) it was derived from

Body size: Extracted from the body mass average column (99).

Habitat breadth: Extracted from columns 115-144 and summed together. We used feeding habitat as a proxy for the habitat most likely used by the species. Column 145 was 'Feeding_habitat_urban', and this was not used as it would have influenced our response variable, the urban tolerance index.

These columns consisted of:

- Feeding_habitat_Terrestrial_Arid_shrubland
- Feeding_habitat_Terrestrial_Chenopod_shrubland
- Feeding_habitat_Terrestrial_Heath
- Feeding_habitat_Terrestrial_Triodia_hummock_grassland
- Feeding_habitat_Terrestrial_Other_grassland
- Feeding_habitat_Terrestrial_Mallee
- Feeding_habitat_Terrestrial_Tropical_savanna_woodland
- Feeding_habitat_Terrestrial_Temperate_dry_sclerophyll_forest_and_woodland
- Feeding_habitat_Terrestrial_Temperate_wet_sclerophyll_forest_and_woodland

- Feeding_habitat_Terrestrial_Rainforest
- Feeding_habitat_Terrestrial_Mangrove_trees
- Feeding_habitat_Inland_waters_Rivers_and_streams
- Feeding_habitat_Inland_waters_Deep_open_water
- Feeding_habitat_Inland_waters_Shallow_open_water
- Feeding_habitat_Inland_waters_Reeds_and_tall_wet_grassland
- Feeding_habitat_Inland_waters_Low_marshland_and_wet_grassland
- Feeding_habitat_Coastal_Sandy
- Feeding_habitat_Coastal_Rocky
- Feeding_habitat_Coastal_Soft_mud
- Feeding_habitat_Coastal_Saltmarsh
- Feeding_habitat_Coastal_Mangrove_floor
- Feeding_habitat_Marine_Cold_pelagic
- Feeding_habitat_Marine_Temperate_pelagic
- Feeding_habitat_Marine_Warm_pelagic
- Feeding_habitat_Marine_Cold_inshore
- Feeding_habitat_Marine_Temperate_inshore
- Feeding_habitat_Marine_Warm_inshore
- Feeding_habitat_Other_non-Australian_habitat
- Feeding_habitat_Agricultural_landscapes

Migratory status – This represented migratory behaviour, either locally dispersing (column 193) or partially migrating (column 194).

These columns consisted of:

- National_movement_local_dispersal
- National_movement_Partial_migrant

A2. Number of eBird checklists for each species prior to and post saptio-temporal subsampling from January 2010-June 2021.

Species with lower than 1,000 checklists with an observation count > 0 post spatio-temporal subsampling were excluded from the analyses.

Species	Number of checklists prior to spatio-temporal subsampling	Number of checklists with an observation count > 0 prior to spatio-temporal subsampling	Number of observations prior to spatio-temporal subsampling	Number of checklists post spatio-temporal subsampling	Number of checklists with an observation count > 0 post spatio-temporal subsampling	Number of observations post spatio-temporal subsampling
Eastern Osprey	608, 756	13,563	19,365	248, 631	9,428	12,792
Black-shouldered Kite	823,966	19,363	26,215	348,979	14,505	19,078
Letter-winged Kite	16,413	39	323	9,647	36	292
Square-tailed Kite	807,516	2,667	3,146	332,119	2,383	2,757
Black-breasted Buzzard	224,304	979	1,253	100,224	924	1,173
Pacific Baza	368,268	5,377	7,580	155,106	4,635	6,517
Black Kite	763,674	41,934	205,488	320,693	28,735	130,283
Whistling Kite	811,101	61,986	105,247	352,609	45,787	76,050
Brahminy Kite	348,685	11,327	15,005	148,187	8,666	11,305
White-bellied Sea-Eagle	804,569	23,780	29,927	339,243	19,651	24,432
Brown Goshawk	840,058	22,330	25,554	360,455	18,551	21,093
Collared Sparrowhawk	828,420	9,495	10,683	345,223	8,411	9,353
Grey Goshawk	738,287	5,474	6,088	290,302	4,677	5,161
Red Goshawk	236,813	86	132	98,333	78	117
Spotted Harrier	807,230	2,779	3,148	332,761	2,496	2,805
Swamp Harrier	821,241	19,405	25,729	344,117	13,520	17,576
Wedge-tailed Eagle	839,024	24,159	35,597	357,597	21,655	31,700
Little Eagle	815,574	5,578	6,333	337,854	4,929	5,574
Nankeen Kestrel	839,347	31,046	40,692	359,812	25,799	33,529
Brown Falcon	839,352	18,237	23,798	354,569	15,604	20,246
Australian Hobby	839,172	11,543	13,181	353,389	9940	11,156

Grey Falcon	235,099	207	342	112,756	185	301
Black Falcon	764,068	1,421	1,775	308, 993	1,305	1,613
Peregrine Falcon	837,301	6,691	7,942	349,365	5,954	7,003
Eastern Barn Owl	840,775	1,273	1,829	348,330	1,051	1,498
Eastern Grass Owl	254,281	235	310	103,445	184	241
Australian Masked Owl	776,041	560	654	309,436	355	413
Sooty Owl	483,370	506	952	165,237	429	542
Lesser Sooty Owl	49,376	260	314	18,628	217	259
Powerful Owl	576,952	3,488	5,603	208,419	2,172	3,287
Rufous Owl	70,579	699	1,067	28,560	429	628
Barking Owl	767,461	1,634	2,666	303,875	1353	2,208
Southern Boobook	816,501	6,724	8,970	339,370	5,477	7,316
Tasmanian Boobook	24,275	271	381	12,317	241	339

A3. Summary statistics of the total number of grids across the focal species distribution

total number of grids across a species distribution with at least one checklist containing an observation of the focal species, the percentage of grids across the focal species distribution containing an observation of the focal species, and the size of the species distribution (km^2). All of these figures are post spatiotemporal subsampling.

Species	Total number of grids across the focal species distribution	Total number of grids across a species distribution with at least one checklist containing an observation of the focal species	Percentage of grids across the focal species distribution containing an observation of the focal species	Size of species distribution (km^2)
Eastern Osprey	11,273	965	8.56	1,844,506
Black-shouldered Kite	23,976	3045	12.70	7,657,212
Letter-winged Kite	1,369	18	1.31	729,216
Square-tailed Kite	22,281	918	4.12	5,957,136
Black-breasted Buzzard	9,952	515	5.17	5,305,882
Pacific Baza	6,373	872	13.68	843,946
Black Kite	20,329	4523	22.25	5,884,718
Whistling Kite	22,097	6291	28.47	5,125,443
Brahminy Kite	5966	902	15.12	1,160,739
White-bellied Sea-Eagle	19,992	2,923	14.62	3,647,386
Brown Goshawk	24,414	3,623	14.85	7,621,370
Collared Sparrowhawk	22,270	2450	11.00	4,733,161
Grey Goshawk	14,952	1,079	7.22	1,805,514
Red Goshawk	4,546	35	0.77	1,075,919
Spotted Harrier	23,158	1294	5.59	7,599,947
Swamp Harrier	22,051	1923	8.72	4,714,167
Wedge-tailed Eagle	24328	6514	26.78	7,686,046
Little Eagle	23,222	1735	7.47	7,271,813
Nankeen Kestrel	24,369	6480	26.59	7,685,869
Brown Falcon	24,336	4950	20.34	7,678,186
Australian Hobby	24,298	2732	11.24	7,676,401
Grey Falcon	11639	98	0.84	5,374,709
Black Falcon	20,042	710	3.54	5,918,750
Peregrine Falcon	23,758	1907	8.03	6,569,133
Eastern Barn Owl	24,448	556	2.27	7,692,945
Eastern Grass Owl	4028	39	0.97	483,699
Australian Masked Owl	18,402	134	0.73	3,138,625
Sooty Owl	5442	132	2.43	338,441
Lesser Sooty Owl	520	35	6.73	55,342

Powerful Owl	8252	307	3.72	573,071
Rufous Owl	932	60	6.44	159,509
Barking Owl	16,525	358	2.17	2,507,900
Southern Boobook	23,455	1941	8.28	7,625,275
Tasmanian Boobook	989	97	9.81	67,371

A4. The median VIIRS values from 1000 random localities in the study region across the years 2014 to 2020.

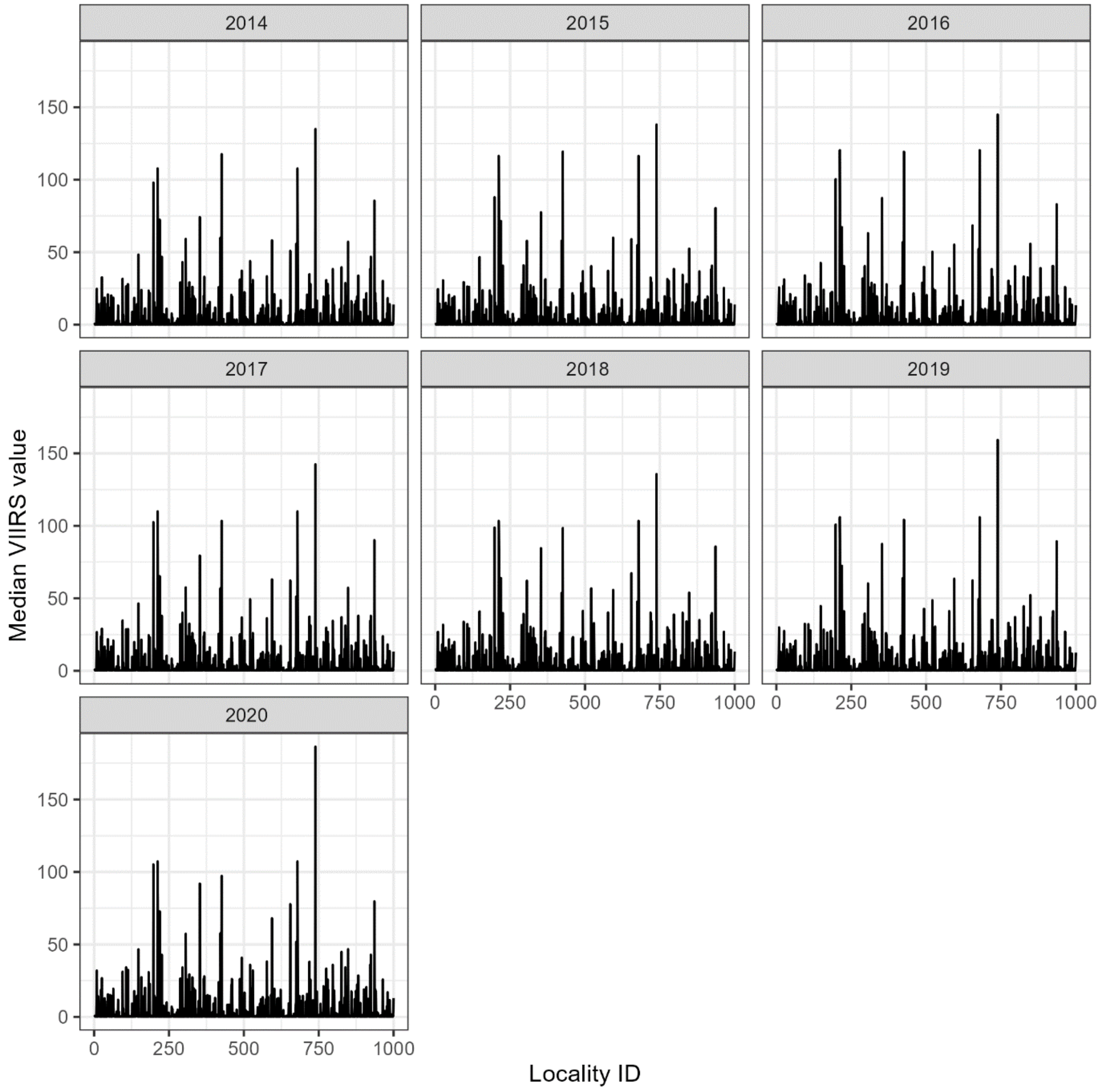

A5. The change in median VIIRS values from 2014 to 2020 from 1000 random localities.

The red line denotes the mean change in the median VIIRS values from 2014 to 2020 for all localities (0.332).

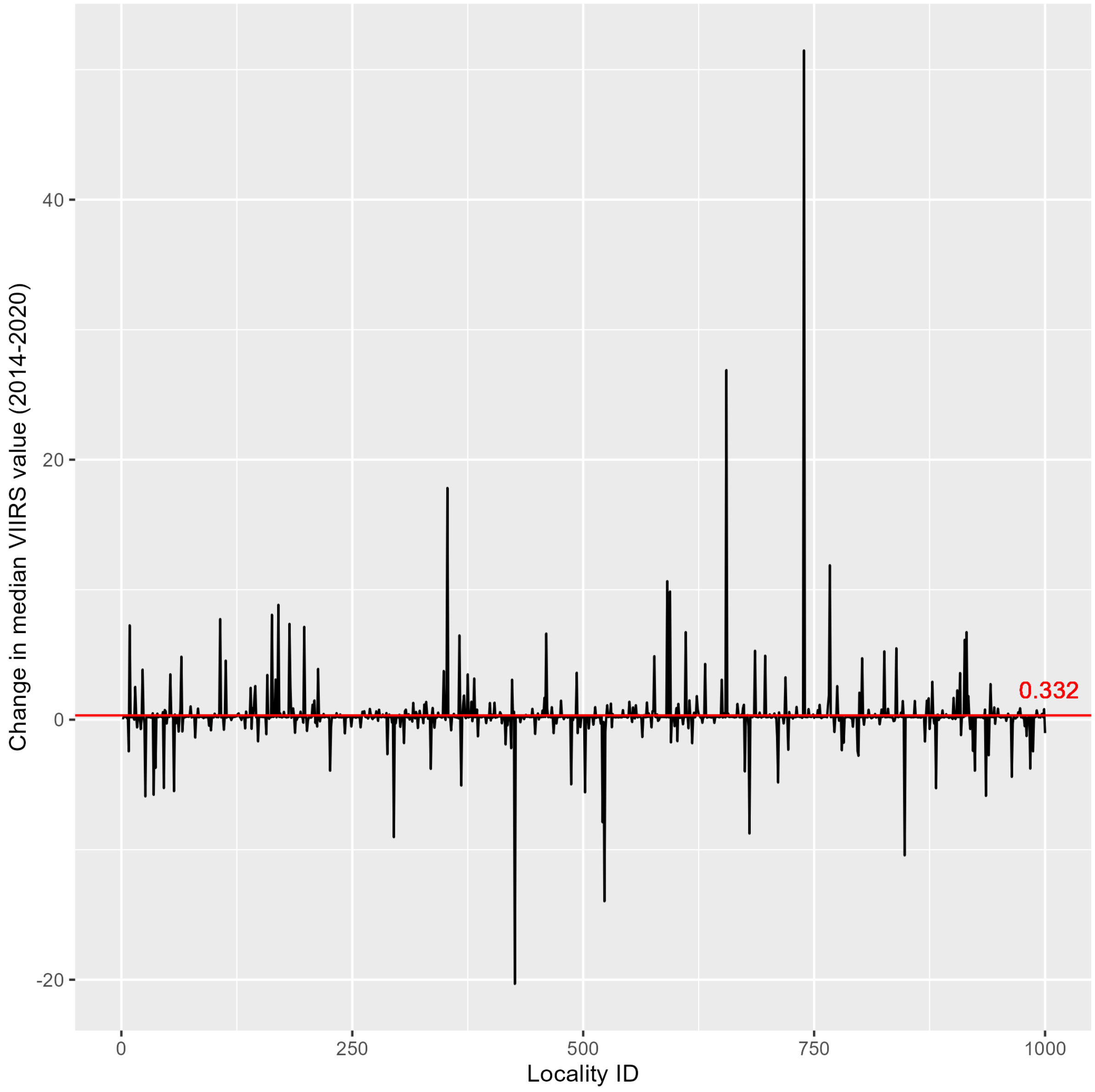

Supplement: Supplementary file 1 — Supplementary Information. [file 41598_2023_38493_MOESM1_ESM.pdf]
